# Buruli Ulcer in United Kingdom Tourist Returning from Latin America

**DOI:** 10.3201/eid1511.090460

**Published:** 2009-11

**Authors:** Hugh McGann, Pieter Stragier, Françoise Portaels, Deborah Gascoyne-Binzi, Timothy Collyns, Sebastian Lucas, Damian Mawer

**Affiliations:** St. James’s University Hospital, Leeds, UK (H. McGann, D. Mawer); Institute of Tropical Medicine, Antwerp, Belgium (P. Stragier, F. Portaels); Leeds General Infirmary, Leeds (D. Gascoyne-Binzi, T. Collyns); St. Thomas’s Hospital, London, UK (S. Lucas)

**Keywords:** Buruli ulcer, Mycobacterium ulcerans, tuberculosis and other mycobacteria, bacteria, Latin America, Brazil, United Kingdom, travel, dispatch

## Abstract

We report a case of Buruli ulcer in a tourist from the United Kingdom. The disease was almost certainly acquired in Brazil, where only 1 case had previously been reported. The delay in diagnosis highlights the need for physicians to be aware of the disease and its epidemiology.

Buruli ulcer (BU) is caused by infection with *Mycobacterium ulcerans* and can lead to extensive destruction of skin and soft tissue (*1*). The disease is endemic in >30 tropical and subtropical countries worldwide (*2*,*3*). It is associated with exposure to stagnant water or slow-flowing rivers. Most cases occur in Africa, and only 1 case has been previously reported from Brazil (*4*). It has rarely been described in travelers returning from an endemic area (*5*,*6*). We report a case of a UK tourist with *M*. *ulcerans* infection after a trip to Brazil and other parts of Latin America.

## The Case

The travel itinerary for this 27-year-old man, his history of water exposure, and the clinical progression of the lesion all support the hypothesis that he acquired the infection in the Pantanal region of southern Brazil. He spent 4 days there starting on August 11, 2007, and participated in trekking on horseback through wetlands and a canoe trip during which he was immersed in water on several occasions. From that region, he flew to the Bolivian cities of Santa Cruz and La Paz, before traveling overland to Lake Titicaca. After 3 days there, he journeyed on to Arequipa, Peru. On September 2, he took a bus trip to the Colca Canyon. During this journey, 17 days after leaving the Pantanal, he first noticed a small, painless papule with an overlying scab on the lateral aspect of his left knee. He had no history of trauma or insect bite and no further water exposure after leaving Brazil.

He then visited Cuzco and some surrounding sites for a week, before entering the rainforests of Manu National Park for a 3-day visit on September 14. Here his leg was immersed in stagnant water on several occasions, although the papular lesion was well established and enlarging by this time. From Cuzco, he went to Lima, the capital of Peru, where he arrived on September 27. When examined in a local clinic 1 week later, the lesion was described as a 1-cm, painless ulcer. Over the next 6 weeks, it gradually increased in size during his travels through Ecuador.

**Figure 1:**
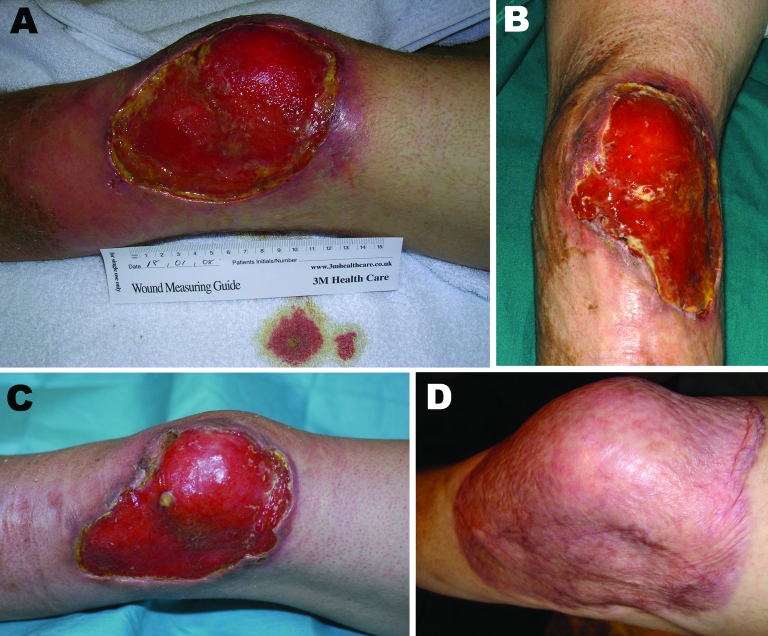
Progression of Buruli ulcer adjacent to the left knee of United Kingdom tourist after returning from Latin America. A) November 2007, on patient’s return to the United Kingdom; B) January 2008, before *Mycobacterium ulcerans* therapy; C) April 2008, after 12 weeks of antimicrobial drug therapy; D) January 2009, 9 months after split-skin grafting.

**Figure 2:**
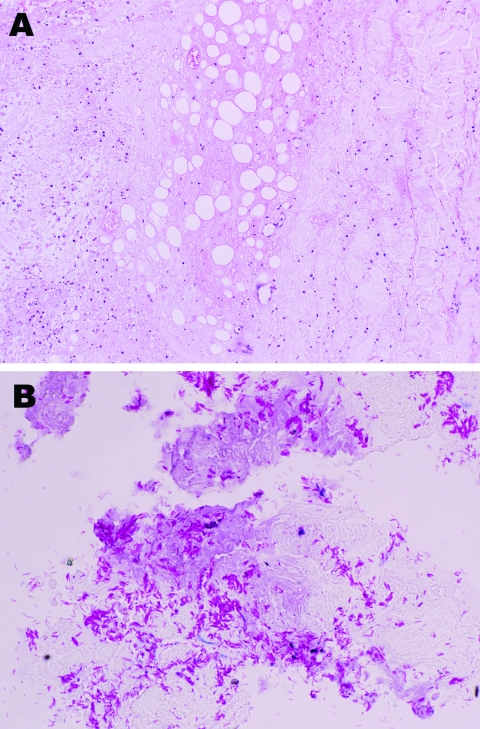
Histologic analysis showing necrosis of subcutaneous fat and deep dermal tissue of the patient. A) Noninflammatory infarction-like necrosis related to cytopathic effect of the mycolactone toxin secreted by *Mycobacterium ulcerans*. B) Abundant mycobacteria within the necrosis.

He returned to the UK on November, 15, 2007, and attended the dermatology department of his local hospital. The ulcer was debrided with larval therapy and measured 11 × 6 cm (Figure 1, panel A). A skin biopsy was performed, and multiple acid-fast bacilli (AFB) were seen on microscopy. Histologic analysis showed necrosis of the subcutaneous fat and deep dermal tissue with clusters of AFB but no granuloma formation (Figure 2). Tissue samples were also sent for mycobacterial culture, but results were negative after 12 weeks incubation. The patient was treated with clarithromycin for presumed *M*. *marinum* infection.

Despite this therapy, the ulcer continued to enlarge, reaching 16 × 10 cm with deeply undermined edges and necrosis of the surrounding skin (Figure 1, panel B). In January 2008, he was referred to the Infection and Travel Medicine Department at our hospital in Leeds. BU was suspected clinically. A swab taken from beneath the ulcer edge was positive for AFB on direct microscopy. A sample was sent to the Institute of Tropical Medicine in Antwerp, Belgium for PCR testing for *M. ulcerans* by detection of the insertion sequence *2404* (IS*2404*), which was positive. Further prolonged cultures for AFB were negative in both the United Kingdom and Belgium.

Because of the extensive nature of the ulcer, the patient was treated for 12 weeks with a daily regimen of 600 mg of oral rifampicin and 400 mg of moxifloxacin. Intravenous amikacin, 15 mg/kg once a day, was added during the first 8 weeks. Response to antimicrobial treatment was satisfactory: the ulcer reduced to 12 × 10 cm, the edges were no longer undermined, and the surrounding skin appeared healthy (Figure 1, panel C). To accelerate healing, split skin grafting was performed 1 month after completion of antimicrobial drug therapy. Nine months later, complete healing had occurred (Figure 1, panel D).

The clinical diagnosis of BU is usually straightforward once the disease has been considered. However, diagnosis may be delayed, as in this case, when the patient is in a country in which BU is not endemic, especially when exposure has occurred in a region where the disease is not well recognized. A major diagnostic advance has been the development of PCR for insertion sequence *2404*, one of 2 multicopy insertion sequences in the *M*. *ulcerans* genome (*7*). The technique, usually performed on tissue biopsy samples, can also be performed directly from ulcer swabs (*8*) and has a sensitivity and specificity of ≈100% (*9*). It is theoretically a rapid test but is not routinely available in many countries, including those where BU is endemic.

## Conclusions

Data from the World Health Organization indicate that the greatest number of BU cases occur in western Africa (*2*). Cases have been reported from the Western Hemisphere, although apart from French Guiana, the disease is considered rare in Central or South America (*3*). For example, in Peru during 1969–2007, only 11 cases were documented (*10*). BU may be endemic in Brazil, but, to our knowledge, only 1 case has been previously reported from this country (*4*).

The use of mycobacterial-interspersed repetitive-unit variable number of tandem repeat typing (MIRU-VNTR) has made it possible to distinguish between different strains of *M. ulcerans* (*11*). Most countries outside Africa have their own unique MIRU-VNTR profile(s). The profile of this patient’s isolate was determined as 10112N, identical to that recovered from a patient from Tumbes in northwest Peru (*10*). This previous case could indicate that our patient acquired his infection in Peru. The epidemiologic evidence, however, supports the hypothesis that it was acquired in the Pantanal region of Brazil, suggesting that this MIRU-VNTR profile has a geographic distribution wider than originally thought.

Cases of BU diagnosed in countries where the disease is not endemic are rare. To our knowledge, only 21 such cases have been reported (*12*). These cases may occur either in a migrant from a country endemic for BU, where the disease is acquired in the country of origin, or in a traveler from a country where BU is not endemic, as in this case. This BU case appears to be only the second reported in a traveler returning from the Western Hemisphere (*12*). Physicians should be aware of its features because early diagnosis and treatment help prevent long-term disability that may result from this infection. Cases such as this one, reported from countries where BU is rare, serve as a reminder that the disease is probably endemic to a larger area than is usually considered.
